# No Spread of SARS-CoV-2 From Infected Symptomatic Children to Parents: A Prospective Cohort Study in a Controlled Hospital Setting

**DOI:** 10.3389/fped.2021.720084

**Published:** 2021-08-03

**Authors:** Francesco Nunziata, Marco Poeta, Edoardo Vassallo, Grazia Isabella Continisio, Andrea Lo Vecchio, Alfredo Guarino, Eugenia Bruzzese

**Affiliations:** ^1^Department of Translational Medical Sciences, Section of Paediatrics, University of Naples Federico II, Naples, Italy; ^2^Department of Neurosciences, University of Naples Federico II, Naples, Italy

**Keywords:** children, severe acute respiratory syndrome coronavirus 2, COVID-19, spreading infection, caregiver

## Abstract

**Introduction:** The transmission rates severe acute respiratory syndrome coronavirus 2 (SARS-CoV-2) from children to adults are unclear due to a lack of controlled conditions.

**Materials and Methods:** We investigated the occurrence of SARS-CoV-2 transmission among 12 discordant child-parent pairs in our ward. In each hospital isolation room, caregivers and children lived in close contact during the entire hospitalization period.

**Results:** A total of 136 swab-positive children (mean age, 3.6 ± 4.9 median age, 1; IQR 0–6.2, range 0.1–17) attended by their caregivers were hospitalized. Of those, 12/136 (8.8%, mean age, 6.1 ± 5.3 median age, 4.5) were attended by caregivers who were swab and serology negative at admission despite previous close contact with positive children at home. Three children were completely dependent on their mothers, one of whom was being breastfed. The mean duration of overall exposure to the index case was 20.5 ± 8.2 days.

**Conclusion:** None of the infected children transmitted SARS-CoV-2 infection to their caregivers, raising the hypothesis of a cluster of resistant mothers or of limited transmission from children to adults despite prolonged exposure and close contact. These data might provide reassurance regarding school openings and offer the chance of investigating SARS-CoV-2 variants in the future under the same quasi-experimental conditions.

## Introduction

SARS-CoV-2 spreading from children is unknown, although school opening is dependent on transmission. At present, children are not included in immunization programs and could be an important and long-lasting source of infection. Korean researchers have shown that transmission from asymptomatic children to adults can occur (1). Although COVID-19 is relatively less severe in children than in adults (2, 3), infants and younger children are believed to actively spread the infection due to the prolonged viral persistence in their respiratory and intestinal tract (4), the lack of social distancing and problems in wearing protective devices, and the need for close contacts by infants and younger children due to feeding and personal hygiene. Scattered data suggest that in

the school setting, SARS-CoV-2 transmission also occurs at low rates due to the use of personal protective devices (5, 6). We aimed to investigate SARS-CoV-2 infection spreading from infected children to adult caregivers in our Pediatric Infectious Disease ward during active pandemic. During this survey (April 2020—February 2021), Italy was under lockdown restriction measures of variable intensity, according to epidemiological data, and there was no solid evidence of SARS-CoV-2 variants spreading. A sensitive and debated measure was related to school opening (7). We admitted 136 children to our reference isolation center for pediatric SARS-CoV-2 infection, which provided a unique opportunity to investigate spreading from children to adults in a strictly controlled setting. Herein, we provide the results of this cohort study.

## Methods

**Figure 1:**
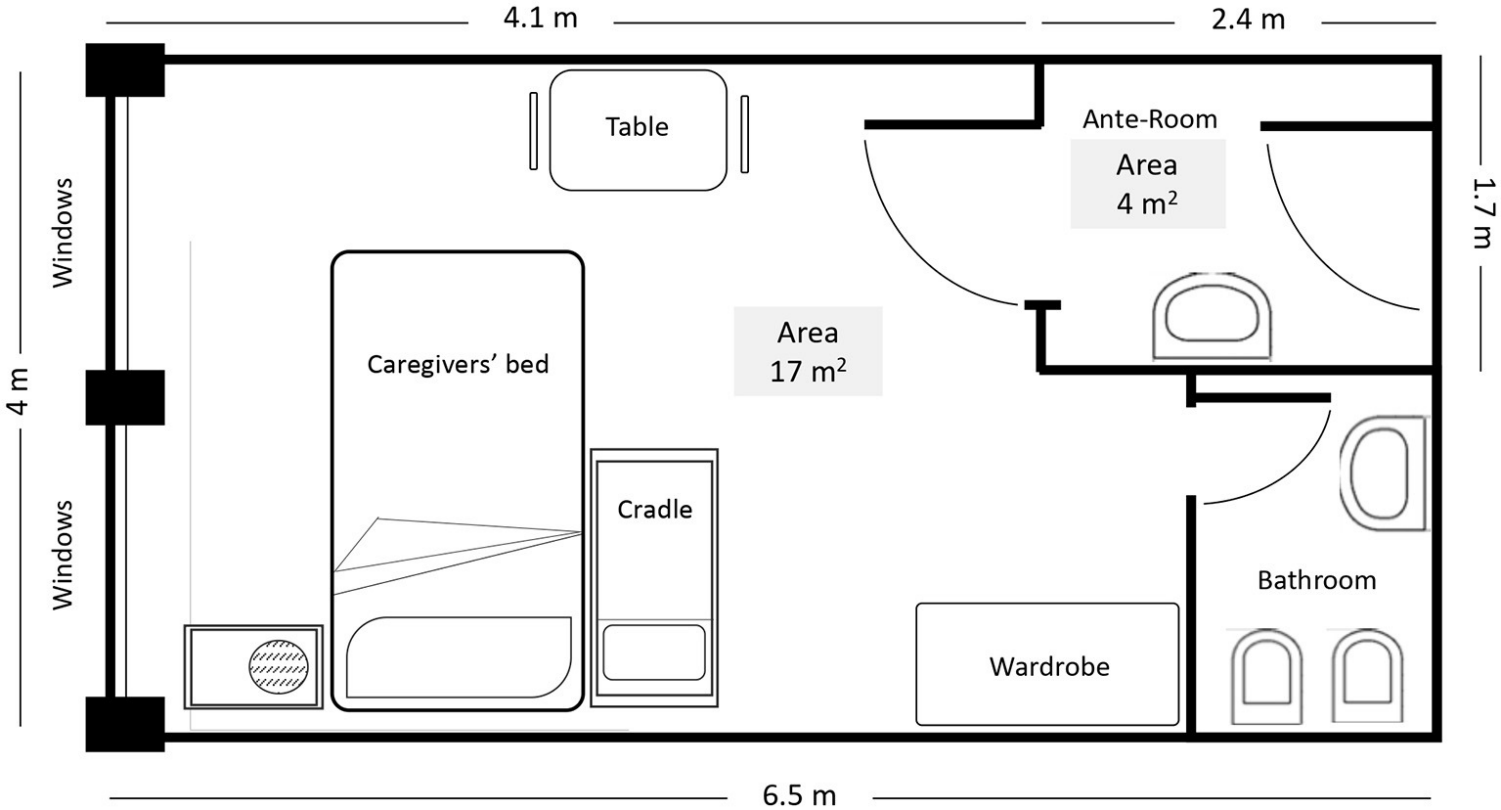
Map of the isolation rooms where the patient and caregiver lived in close contact.

This is a prospective cohort study evaluating SARS-CoV-2 infection spreading from infected children to their non-infected caregivers admitted to our ward. All admitted discordant pairs were considered eligible for our study. The ward has an area dedicated to SARS-CoV-2-infected patient care with eight isolation rooms. Each room has an area of 17 square meters and includes a small bathroom. The room has two windows but no negative pressure, and its access has a filter space (Figure 1). In each room, the caregiver and the child were living in close contact during the entire hospitalization. Child feeding, diaper and clothing changes, oral therapy administration, and child hygiene were provided by the caregiver (8), who was also asked to wear a surgical mask and to ensure hand hygiene and routine cleaning of surfaces with antiseptics. Both the child and caregiver were swab tested at admission and every 3–5 days, depending on clinical conditions, until virus clearance and subsequent hospital discharge were achieved. In some cases, children in good clinical conditions were discharged when swab was still positive and caregivers were swabbed at home. To evaluate the spread of infection, the cycle threshold (C_t_) from real-time (quantitative) reverse transcription polymerase chain reaction (qPCR) of SARS-CoV-2 swab-positive infants was also obtained (9). An anti-SARS-CoV-2 IgG assay was performed by electrochemiluminescence at hospital admission, after 14, 21 days and 1, 3 months after discharge. To define the overall exposure to SARS-CoV-2, an accurate clinical anamnestic interview was obtained for each pair. The mean duration of exposure was calculated starting from the day of symptom onset and/or the time of first swab positivity until the first evidence of swab negativity. The protocol of our study was approved by the ethics committee of University Federico II of Naples, Italy (protocol No. 261/20).

## Results

A total of 136 swab-positive children (mean age, 3.6 ± 4.9 years; median age, 1 years; IQR 0–6.2, range 0.1–17) attended by their caregivers (133 mothers and three fathers) were hospitalized. A total of 104 were in mild clinical conditions (76%), whereas 20 (14%), 11 (8%) and 1 (0.7%) were in moderate, severe and critical conditions, respectively.

A total of 12/136 (8.8%) caregivers were swab and serology negative at admission despite previous close contact with positive children at home. In all cases personal contacts with SARS-CoV-2 were reported, five of which were housemates. Cohabitation was even closer than usual due to lockdown restraints. The serological and epidemic features of the 12 discordant pairs (positive patient/negative caregiver) are shown in Table 1.

**Table 1:** Serological and epidemic features of patients and caregivers.

	**Patients**	**Caregivers**
Mean age (yrs)	6.1 ± 5.3	34.8 ± 7.4
Gender (F) *n*; %	9/12 (75)	9/12 (75)
Mask wearing *n*; %	5/12 (41)	12/12 (100)
Positive SARS-CoV-2 IgG *n*; %	8/12 (66)	0/12 (0)
Positive SARS-CoV-2 IgG (after 1–3 months) *n*; %	12/12 (100)	0/12 (0)
Mean days of hospitalization	9.2 ± 7.4	9.2 ± 7.4
Days of exposure to patients (overall exposure)	-	20.5 ± 8.2

The mean age of the children was 6.1 ± 5.3 years, and the median age was 4.5 years. A total of 3/12 (25%) children were totally dependent on their caregivers for all feeding and hygiene needs. Seven were able to wear masks, but in most cases, this was not always the case. We estimate that masks were used for a maximum of 50% of the total time and not always in a suboptimal wearing efficiency. One infant was exclusively breastfed. In most cases, children were in mild to moderate clinical conditions, but one child required oxygen administration, and three received intravenous therapy. Three of the 12 children had a substantial clinical problem concurrently with SARS-CoV-2 infection, namely sepsis, renal colic and diabetes, whereas two child had COVID-19 pneumonia and another one presented with mild respiratory symptoms (cough). Three of the 12 children had underlying chronic conditions (very low birth weight infant with heart disease and bronchopulmonary dysplasia, cystinosis, and cerebral palsy). The initial evidence of infection in a positive close contact was identified in all cases based on symptom onset or swab positivity and preceded the child diagnosis by 7–15 days, pointing toward infection initial spreading from an adult to the child rather than the opposite within the housemates.

**Figure 2:**
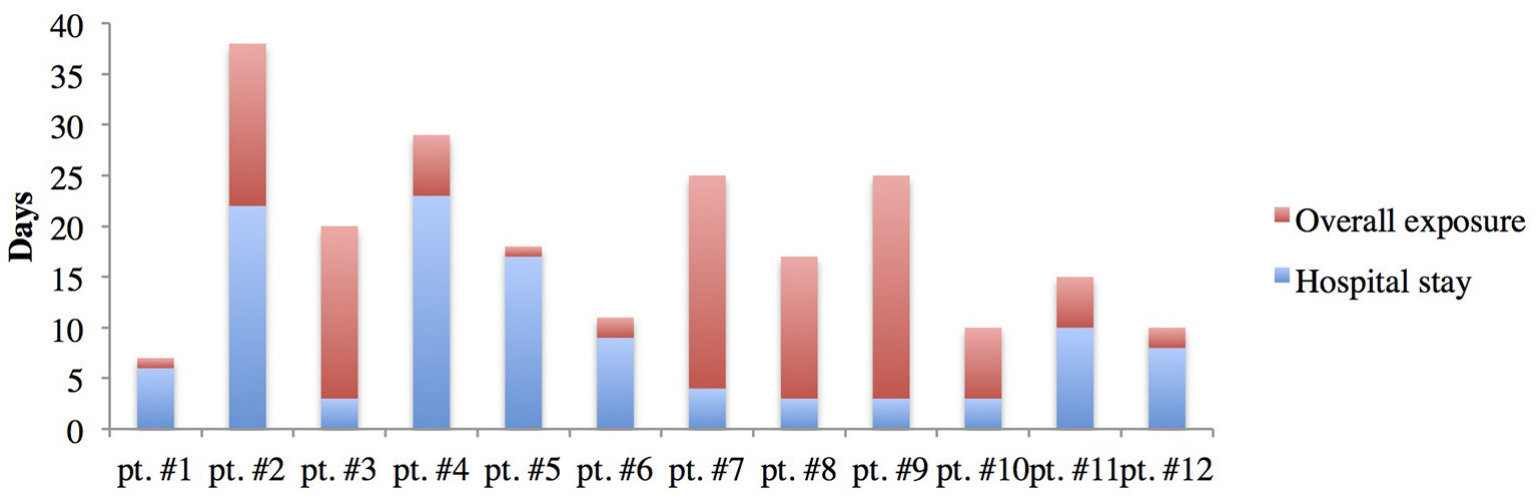
Overall and in-hospital time of exposure of each caregiver to his/her SARS-CoV-2-infected child.

Caregivers were three fathers and eight mothers. Their mean age was 34.8 ± 7.4 years, and their median age was 37. All caregivers adhered quite well to clinical indications about safety and restriction measures consequent to hospital isolation. The mean duration of hospitalization was 9.2 ± 7.4 days (range, 3–23). Based on the clinical history of contacts, we estimated that the mean total time of SARS-CoV-2 exposure, including prehospital admission, lasted 20.5 ± 8.2 days (range, 7–38). The total and in-hospital times of exposure of discordant pairs are shown in Figure 2. In five cases, children were discharged while still positive. Serology was positive in eight children during hospitalization.

All caregivers performed a serological test to evaluate the presence of specific anti SARS-COV-2 antibodies 1 and 3 months after viral clearance of their child and all resulted negatives. This finding confirmed that SARS-CoV-2 was not transmitted from the child to the caregiver in any of our 12 cases, despite prolonged hospitalization, continuous close contact and C_t_ in an half of patients below 24 cycles (32, 24, 20, 9, 36, 6, 12, 30, 8, 32, 30, 27), indicating active viral replication (10). Anti SARS-COV-2 antibodies were evaluated again in 6/12 caregivers 6 months after discharge and in two of them were found positive.

## Discussion

Concerns have been raised that familial and social contacts involving children, including nurseries and school activities, could facilitate SARS-CoV-2 spread (11). In a recent survey in an English primary school setting, infection rates were low in asymptomatic children (12). A recent commentary raises a number of unresolved issues associated with school reopening. This was carried on with different approaches and times, worldwide with major discrepancies related to the risk of infection transmission based on previous pandemics (7). Our data, although limited by small sample size, challenge the strategy of strict school closure also in the light of the slow severity of COVID 19 in children. In addition, children frequently did not have respiratory symptoms responsible for viral spreading.

Owing to the peculiar setting, we had the opportunity to survey infection spreading from children to adults under strictly controlled conditions. Our results were highly consistent and reassuring: no evidence of transmission from children to caregivers was documented in the hospitalized setting, despite couples living in a relatively small area with limited toilet facilities. All 12 caregivers remained healthy and uninfected after close contact for more than 1 week on average. Potential explanations include a limited viral load (which would be denied by available data of a sustained viral load in children) or a specific behavior of the virus coming from a child or-conversely- with the efficacy of limiting social contacts while in a hospital setting. The latter is supported by a previous study performed in our institution showing no transmission among health workers due to the strict containing measures applied to prevent infection spreading (13).

Considering that all the caregivers wore surgical masks during the hospital stay, we can hypothesize that the surgical mask may be a critical means against the transmission of infection. Although the sample is limited, it is somehow surprising that in no case was SARS-CoV-2 transmitted from the child to the adult caregiver notwithstanding a continuous close cohabitation. This conflicts with the considerations of spreading routes that generally involve children and include proximity, close dependency and contact with at-risk adults. Whether these reflect an unknown resistance to infection in selected population subsets is currently unknown. Recruited caregivers may have personal factors that do not allow SARS-CoV-2 infection as demonstrated in HIV infection where genetic and epigenetic factors are involved in natural resistance to infection (14). Further investigations are needed to evaluate these factors. Considering that only three children had respiratory involvement, we can suppose that the absence of respiratory symptoms may represent an additional factor for a low transmission of the infection from children to their caregivers. In conclusion, our results have been obtained in a peculiar well-controlled condition and, although limited in numbers, are very consistent. They suggest that children are not a major source of spreading, which is reassuring in light of the present policy of putting children on a very low priority scale in immunization programs. We cannot rule out that new viral mutants may change this pattern in the near future. Therefore, further data are needed in order to estimate the transmission rates from children to adults, the correlation of clinical features to the risk of transmission in pediatric settings and the efficacy of barriers to limit SARS-CoV-2 spreading. A better understanding of the pathophysiology of SARS-CoV-2 infection in children, including the risk of transmission from child to adults, would reduce the social impact of infection.

## Data Availability Statement

The raw data supporting the conclusions of this article will be made available by the authors, without undue reservation.

## Ethics Statement

The studies involving human participants were reviewed and approved by protocol No. 261/20 University Federico II of Naples, Italy. Written informed consent to participate in this study was provided by the participants' legal guardian/next of kin.

## Author Contributions

AG conceptualized and designed the study and reviewed and revised the manuscript. FN, MP, EV, GC, AL, and EB collected data, performed the initial analyses, drafted the initial manuscript, and reviewed and revised the manuscript. All authors approved the final manuscript as submitted and agree to be accountable for all aspects of the work.

## Conflict of Interest

The authors declare that the research was conducted in the absence of any commercial or financial relationships that could be construed as a potential conflict of interest.

## Publisher's Note

All claims expressed in this article are solely those of the authors and do not necessarily represent those of their affiliated organizations, or those of the publisher, the editors and the reviewers. Any product that may be evaluated in this article, or claim that may be made by its manufacturer, is not guaranteed or endorsed by the publisher.
